# Exercise-Induced Circulating Lactate Responses in Breast Cancer Survivors: A Systematic Review and Exploratory Meta-Analysis

**DOI:** 10.3390/muscles5030047

**Published:** 2026-07-02

**Authors:** Amir Hossein Ahmadi Hekmatikar, Gema Santamaría, Ana M. Celorrio San Miguel, Enrique Roche, Fatemeh Khodadadi, Álvaro López-Llorente, Diego Fernández-Lázaro

**Affiliations:** 1 Department of Physical Education and Sport Sciences, Faculty of Humanities, Tarbiat Modares University, Tehran 10600, Iran; 2 Department of Anatomy and Radiology, Faculty of Health Sciences, Campus of Soria, University of Valladolid, 42004 Soria, Spain; 3 Neurobiology Research Group, Faculty of Medicine University of Valladolid, 47005 Valladolid, Spain; 4 Department of Applied Biology-Nutrition, Institute of Bioengineering, Miguel Hernández University, 03202 Elche, Spain; 5 Alicante Institute for Health and Biomedical Research (ISABIAL), 03010 Alicante, Spain; 6 CIBER Physiopathology of Obesity and Nutrition (CIBEROBN), Carlos III Health Institute (ISCIII), 28029 Madrid, Spain; 7 Department of Exercise Physiology, Ferdowsi University of Mashhad, Tehran 10600, Iran; 8 Department of Sports Medicine, Burgos Burpellet BH UCI Pro Team, 09007 Burgos, Spain; 9 Area of Histology, Faculty of Health Sciences, Campus of Soria, University of Valladolid, 42004 Soria, Spain; 10 Biomedical Research Institute of Leon (IBIOLEÓN), University Hospital Complex of Leon, 24071 Leon, Spain

**Keywords:** breast cancer survivors, exercise physiology, blood lactate, metabolic adaptation, cancer rehabilitation, systematic review, meta-analysis, exercise metabolism

## Abstract

**Background:** Physical exercise is strongly recommended for breast cancer survivors due to its beneficial effects on physical function, metabolic health, and quality of life. Lactate, traditionally considered a metabolic byproduct of glycolysis, is increasingly recognized as a signaling molecule involved in metabolic regulation and exercise adaptation. However, exercise-induced circulating lactate responses in breast cancer survivors remain poorly characterized. The aim of this systematic review and exploratory meta-analysis was to synthesize and critically appraise current evidence on exercise-induced circulating lactate responses in breast cancer survivors **Methods:** A systematic review and exploratory meta-analysis were conducted according to PRISMA guidelines and prospectively registered in PROSPERO (CRD42024504288). PubMed, Scopus, and Web of Science were searched to identify controlled trials investigating exercise-induced changes in circulating lactate concentrations in breast cancer survivors. Random-effects meta-analysis was performed using pooled mean differences. **Results:** Among 173 screened records, four studies met eligibility criteria for qualitative synthesis and three contributed to quantitative analysis. Pooled results demonstrated no statistically significant effect of exercise on circulating lactate concentrations (weighted mean difference: 0.03 mmol/L; 95% CI: −0.24 to 0.31; *p* = 0.81), with low heterogeneity (I^2^ = 31.1%). Considerable variation was observed across exercise protocols, intervention duration, and lactate assessment timing. **Conclusions:** Exercise-induced circulating lactate responses in breast cancer survivors appear modest and inconsistently reported across available studies. Current evidence remains limited by small sample sizes and methodological heterogeneity. These findings provide a physiological overview of lactate responses to exercise in breast cancer survivorship and highlight the need for standardized exercise interventions and metabolic outcome assessment in future research.

## 1. Introduction

Breast cancer remains the most frequently diagnosed cancer among women worldwide, with a growing population of survivors experiencing long-term physiological, metabolic, and functional challenges following treatment [1]. The increasing number of breast cancer survivors represents a growing clinical and economic challenge for healthcare systems worldwide due to long-term treatment-related complications, reduced physical function, fatigue, and decreased quality of life. Rehabilitation strategies aimed at improving functional capacity and reducing treatment-related adverse effects have therefore become increasingly important in survivorship care. Among these strategies, structured physical exercise has emerged as a cost-effective and evidence-based supportive intervention. Exercise is widely recommended as a non-pharmacological strategy to improve cardiometabolic health, physical capacity, fatigue, and quality of life in breast cancer survivors [2]. Exercise interventions have also been associated with improvements in muscular strength, cardiorespiratory fitness, cancer-related fatigue, psychological well-being, and treatment tolerance in breast cancer survivors. In addition, regular physical activity may contribute to reduced physical deconditioning and improved functional independence following surgery, chemotherapy, or radiotherapy. These physiological and psychosocial benefits have positioned exercise as an important supportive strategy in cancer rehabilitation and survivorship care [2]. Despite the established benefits of exercise in cancer rehabilitation, the metabolic responses elicited by exercise in this population remain incompletely characterized.

Lactate is a transient metabolic product generated during glycolytic energy production, particularly during moderate- to high-intensity exercise. Traditionally regarded as a byproduct of anaerobic metabolism, lactate is now recognized as an important signaling molecule involved in intercellular communication and metabolic regulation [3,4,5]. During exercise, circulating lactate concentrations may reflect substrate utilization, exercise intensity, and metabolic adaptation, making lactate a physiologically relevant marker of exercise response [6,7,8,9]. During high-intensity exercise, lactate accumulation has traditionally been associated with muscular fatigue, reduced exercise tolerance, and metabolic acidosis, although contemporary evidence indicates that lactate itself is not the direct cause of fatigue. Instead, circulating lactate reflects the balance between production, clearance, and tissue utilization during exercise. Exercise training may improve lactate transport, oxidation, and clearance capacity through adaptations in mitochondrial function, oxidative enzyme activity, and monocarboxylate transporter expression. Consequently, lactate dynamics may provide indirect insight into metabolic efficiency, exercise tolerance, and physiological adaptation in clinical populations [10,11,12,13].

In cancer research, elevated lactate concentrations within the tumor microenvironment have been associated with altered cellular metabolism, immune regulation, and angiogenic signaling [10,11,12,13,14,15,16,17]. However, much of this evidence derives from tumor tissue, experimental models, or pre-clinical investigations. These findings cannot be directly extrapolated to transient changes in circulating blood lactate measured during exercise in clinical populations.

Exercise-related lactate responses may be particularly relevant in breast cancer survivors due to treatment-related alterations in metabolic function, physical deconditioning, and reduced exercise tolerance. Nevertheless, the available evidence examining circulating lactate responses to exercise in breast cancer survivors remains limited, fragmented, and methodologically heterogeneous. Previous studies have evaluated different exercise modalities, intensities, and lactate assessment protocols using relatively small samples, which limits comparability across findings and complicates interpretation of physiological responses. To date, no systematic review or meta-analysis has specifically synthesized the available evidence regarding exercise-induced circulating lactate responses in breast cancer survivors.

For clinicians involved in exercise prescription and cancer rehabilitation, understanding physiological responses to exercise may help contextualize metabolic adaptation in breast cancer survivors. However, the extent to which exercise influences circulating lactate in this population remains unclear. Therefore, the aim of this systematic review was to synthesize and critically appraise the available evidence regarding exercise-induced changes in circulating blood lactate in breast cancer survivors through different types of exercise, with a physiological and descriptive focus.

## 2. Results

### 2.1. Study Selection

The database search identified 173 records. After removal of 48 duplicate articles and exclusion of 115 records based on title and abstract screening, 10 full-text articles were assessed for eligibility.

Among these, one study was excluded due to duplicate reporting from the same dataset, with the most comprehensive publication retained for analysis. Additional exclusions included two studies involving prostate cancer comparator groups, one study that did not report lactate outcomes, and two studies lacking an exercise intervention.

Ultimately, four randomized controlled trials met the eligibility criteria for inclusion in the qualitative synthesis. Of these, three provided sufficient quantitative data for inclusion in the meta-analysis, while one study was excluded from quantitative synthesis because variability measures required for effect size estimation were not reported.

**Figure 1:**
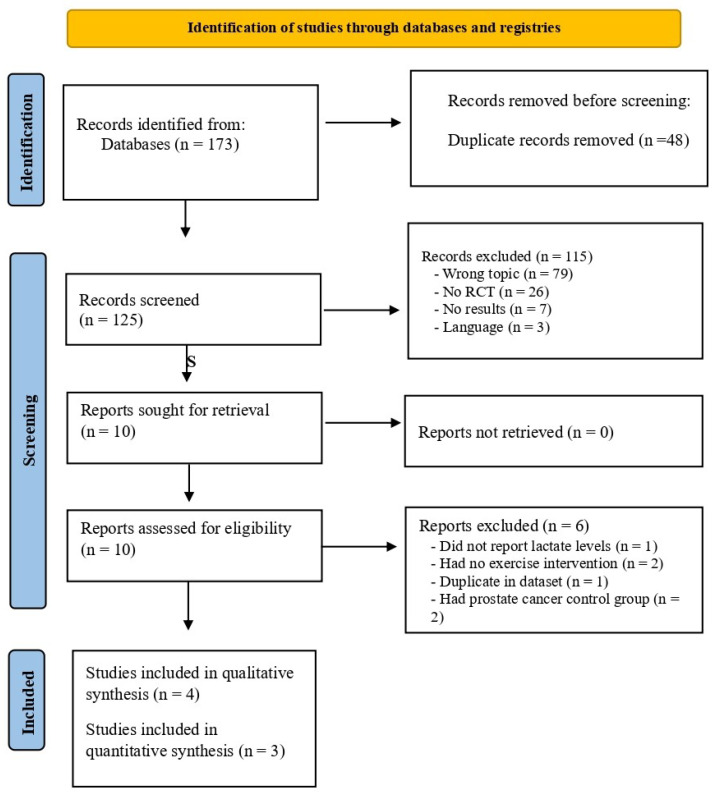
PRISMA flow diagram illustrates the study identification, screening, eligibility, and inclusion process.

The study selection process is summarized in Figure 1.

### 2.2. Assessment of Methodological Quality

Methodological quality assessed using the McMaster Critical Review Form for Quantitative Studies indicated that all included trials met acceptable quality standards (Table 1).

According to the McMaster assessment, two studies demonstrated good methodological quality [18,19], one showed very good methodological quality [20], and one demonstrated excellent reporting and methodological characteristics according to the scale criteria [21]. Common limitations included incomplete reporting of blinding procedures and limited sample size justification.

**Table 1:** Methodological quality assessment of included studies using the McMaster Critical Review Form for Quantitative Studies.

Study, Year	Item																Total	%	Quality Score
	1	2	3	4	5	6	7	8	9	10	11	12	13	14	15	16			
Tosti et al. (2011) [18]	1	1	0	1	1	0	1	1	0	1	0	1	0	1	1	1	11	68.75	G
Neil et al. (2013) [20]	1	1	1	1	1	1	1	1	1	1	0	1	1	1	1	0	14	87.5	VG
Evans et al. (2016) [19]	1	1	0	1	1	0	1	1	0	1	0	1	0	1	1	1	11	68.75	G
Hiraoui et al. (2019) [21]	1	1	1	1	1	1	1	1	1	1	1	1	1	1	1	0	15	93.8	E

Abbreviations: E, excellent; VG, very good; G, good. Detailed item descriptions are available in the original McMaster Critical Review Form publication [22].

Assessment using the PEDro scale indicated generally high methodological quality across included studies (Table 2).

Two studies achieved PEDro scores of 8, indicating good methodological characteristics according to the PEDro scale [18,19], while two studies scored 9, reflecting high PEDro-assessed quality [20,21]. Therefore, PEDro, McMaster, and Cochrane risk-of-bias assessments were interpreted as complementary rather than interchangeable methodological evaluations. Overall, methodological assessment suggested generally adequate reporting quality regarding study design, intervention description, outcome measurement, and statistical analysis across included trials, although reporting quality should not be interpreted as equivalent to low risk of bias or strong internal validity.

Limitations were primarily related to the lack of blinding of participants, therapists, and assessors, which is common in exercise intervention studies.

### 2.3. Risk-of-Bias Assessment

Risk-of-bias assessment was conducted using the Cochrane risk-of-bias framework for randomized trials [24]. Overall, methodological quality varied across studies, with two trials classified as having low risk of bias and two presenting higher levels of methodological concern.

The most frequent sources of bias were related to missing outcome data, outcome measurement procedures, and selective reporting of results. Concerns regarding deviations from intended interventions and incomplete blinding procedures were also observed in some studies.

**Figure 2:**
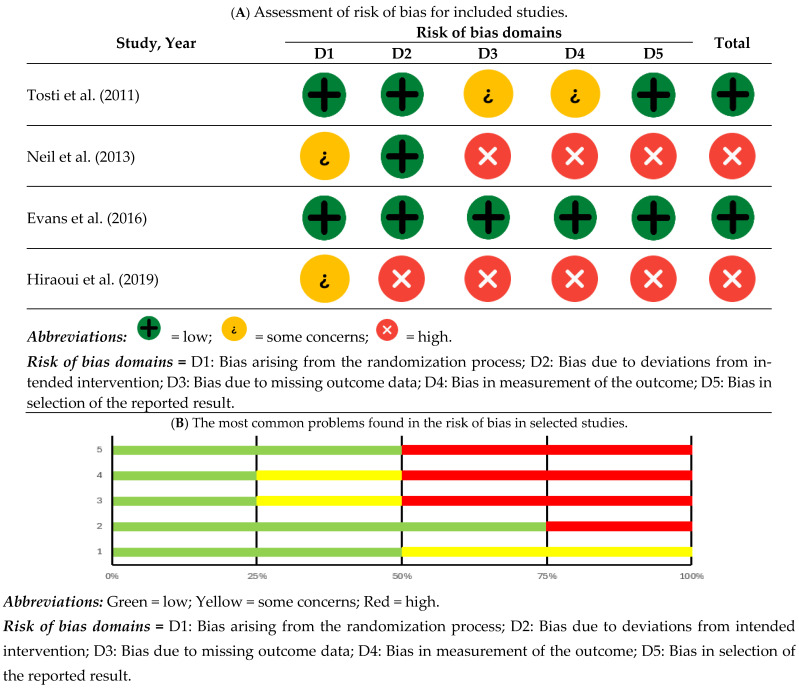
Risk-of-bias assessment of included randomized controlled trials. (**A**) illustrates overall risk-of-bias judgments across domains, while (**B**) summarizes the proportion of studies classified as low risk, some concerns, or high risk for each domain. References: Tosti et al. (2011) [18], Neil et al. (2013) [20], Evans et al. (2016) [19], Hiraoui et al. (2019) [21].

A detailed summary of risk-of-bias assessments across individual domains is presented in Figure 2.

### 2.4. Characteristics of Included Studies

Characteristics of the included studies are summarized in Table 3A,B.

Four randomized controlled trials published between 2011 and 2019 met the eligibility criteria for qualitative synthesis. Three studies provided sufficient quantitative data for inclusion in the meta-analysis.

Across all included trials, a total of 117 participants were enrolled, including 67 individuals in exercise intervention groups and 50 controls. Participants were predominantly middle-aged breast cancer survivors, with mean ages ranging from approximately 49 to 57 years. Cancer stages were generally early to locally advanced (stages I–III/IIIA).

Most interventions evaluated acute exercise responses, with three studies employing single-session cycle ergometer protocols [18,19,20]. One study investigated chronic exercise adaptation using a supervised intermittent cycling program conducted twice weekly over six weeks [21].

Comparator groups varied across studies and included healthy women, breast cancer survivors without persistent fatigue, and non-exercise controls. Exercise protocols differed in intensity and duration, contributing to clinical heterogeneity among included trials.

### 2.5. Findings from the Systematic Review

Baseline, post-intervention, and change values for circulating lactate concentrations are summarized in Table 4.

Across the four included trials, exercise-induced lactate responses showed substantial variability. Two studies reported attenuated lactate responses in breast cancer survivors compared with comparator groups [18,20], whereas one study observed increases in lactate concentrations following acute exercise [19]. The remaining study, which evaluated a chronic exercise intervention, reported reductions in circulating lactate following six weeks of supervised training [21].

Overall, acute exercise protocols generally produced transient increases in lactate concentrations, although the magnitude of response varied according to exercise intensity, comparator population, and measurement timing. In contrast, chronic exercise exposure appeared to be associated with reduced resting lactate levels, suggesting possible metabolic adaptation.

Despite these observations, findings remained heterogeneous across studies due to differences in intervention characteristics, comparator groups, and lactate assessment protocols.

### 2.6. Findings from the Meta-Analysis

Three randomized controlled trials [18,19,20] provided sufficient quantitative data and were included in the meta-analysis, comprising a total sample of 59 participants.

**Figure 3:**
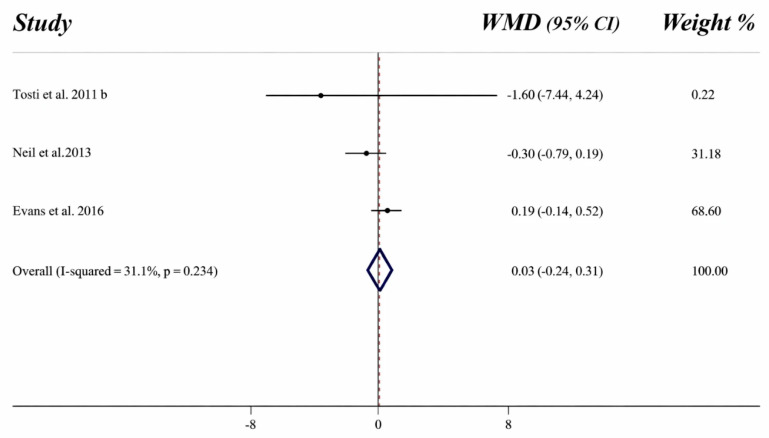
Forest plot showing weighted mean differences (WMDs) and 95% confidence intervals for the effect of exercise interventions on circulating lactate concentrations in breast cancer survivors. Abbreviations: CI, confidence interval; WMD, weighted mean difference. References: Tosti et al. (2011) [18], Neil et al. (2013) [20], Evans et al. (2016) [19].

The pooled analysis showed no statistically significant effect of exercise on circulating lactate concentrations in breast cancer survivors (weighted mean difference [WMD]: 0.03 mmol/L; 95% confidence interval [CI]: −0.24 to 0.31; *p* = 0.813) (Figure 3).

Between-study heterogeneity was low (I^2^ = 31.1%, *p* = 0.234), suggesting moderate consistency across included studies despite differences in intervention characteristics and comparator groups.

Sensitivity analyses using a leave-one-out approach demonstrated that pooled estimates remained directionally stable following sequential exclusion of individual studies. Additional analyses using alternative pre–post correlation coefficients (r = 0.5 and r = 0.7) produced overlapping confidence intervals, indicating limited sensitivity to assumptions regarding SD-change estimation.

Given the small number of included studies and variation in exercise protocols, findings should be interpreted as exploratory and descriptive rather than definitive estimates of effect.

Formal assessment of publication bias was not performed because fewer than 10 studies were included in the meta-analysis, rendering funnel plot asymmetry tests unreliable and potentially misleading.

### 2.7. Leave-One-Out Sensitivity Analysis

A leave-one-out sensitivity analysis was performed to evaluate the influence of individual studies on the pooled effect estimate. Each study was sequentially removed, and the meta-analysis was repeated to determine whether any single trial disproportionately affected the overall findings.

**Figure 4:**
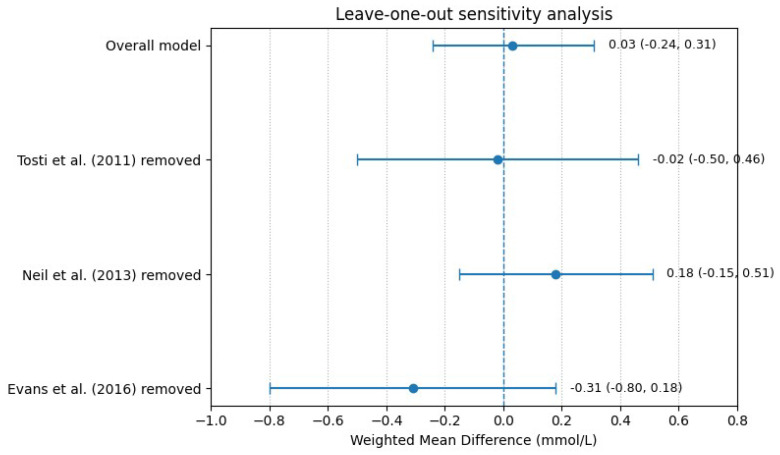
Leave-one-out sensitivity analysis showing pooled weighted mean differences after sequential exclusion of each study. Note: Horizontal lines represent 95% confidence intervals for pooled estimates obtained after removing individual studies. References: Tosti et al. (2011) [18], Neil et al. (2013) [20], Evans et al. (2016) [19].

The pooled estimates remained relatively stable across all iterations, indicating that no individual study substantially influenced the direction or magnitude of the overall effect (Table 5; Figure 4). Although confidence intervals widened when specific studies were excluded, the pooled estimates consistently crossed the null value, supporting the robustness of the primary findings.

These results suggest that the absence of a statistically significant pooled effect was not driven by a single study but reflected the overall consistency of the limited available evidence [25].

## 3. Discussion

The present systematic review and meta-analysis provides a structured synthesis of the limited available evidence on exercise-induced changes in circulating blood lactate in breast cancer survivors. Rather than advancing clinical recommendations, the purpose of this synthesis is to critically define what can—and cannot—be inferred from the existing literature. Across four randomized controlled trials eligible for qualitative synthesis, and three trials contributing to the quantitative analysis, no statistically significant pooled effect of exercise on circulating lactate levels was observed. These findings should be interpreted as a physiological summary of reported lactate responses rather than as evidence supporting or refuting oncologic safety, risk, or clinical benefit [18,19,20,21].

Before interpreting the findings, it is essential to emphasize that the present synthesis is based on a very small number of randomized trials with limited sample sizes and heterogeneous exercise protocols. Moreover, the outcome of interest—circulating blood lactate—represents a short-term systemic physiological response to exercise rather than a validated oncologic endpoint [6,7,8,9]. Consequently, the findings should be interpreted as exploratory and descriptive rather than confirmatory.

Importantly, the present review does not evaluate cancer recurrence, metastatic risk, tumor progression, or oncologic safety. Circulating lactate cannot be interpreted as a surrogate marker of tumor behavior or clinical cancer outcomes [10,11,12,13,14,15,16,17]. None of the included trials assessed tumor-specific lactate concentrations or oncologic endpoints. Accordingly, the absence of a statistically significant pooled effect should not be interpreted as evidence of either oncologic risk or protection, nor as a basis for clinical decision making regarding breast cancer prognosis.

Overall, the findings indicate that exercise did not exert a statistically significant effect on circulating lactate concentrations in breast cancer survivors. Importantly, none of the included studies directly examined the relationship between lactate and tumor growth, nor did they assess tumor-specific metabolic responses. Consequently, whether exercise-induced systemic lactate fluctuations have any relevance to tumor metabolism or progression remains unknown [10,11,12,13,14,15,16,17].

These considerations are clinically relevant for healthcare professionals involved in exercise prescription and cancer rehabilitation, including sports medicine specialists, physiotherapists, and exercise physiologists. Exercise remains an established supportive intervention for improving physical function and quality of life in breast cancer survivors [2]; however, current evidence does not support interpretation of circulating lactate as a clinically meaningful oncologic biomarker.

### 3.1. Clinical Relevance of Blood Lactate as an Outcome

Blood lactate reflects a transient systemic metabolic response to exercise and is not a validated surrogate marker for breast cancer recurrence, metastasis, or tumor progression [10,11,12,13,14,15,16,17]. The biological roles attributed to lactate in cancer largely derive from studies of intratumoral lactate accumulation and tumor microenvironment dynamics, which occur under spatial and concentration conditions that differ substantially from short-lived changes measured in peripheral blood [10,11,12,13,14,15,16,17].

The present findings therefore provide a physiological characterization of exercise-related lactate responses in breast cancer survivors [3,4,6,7]. Although exercise-induced lactate fluctuations may be biologically relevant from a metabolic perspective, there is currently no evidence demonstrating that these transient systemic changes translate into alterations in tumor metabolism, recurrence risk, or cancer progression.

### 3.2. Interpretation of Exercise-Induced Lactate Responses

Among the included studies, three trials [18,19,20] examined acute exercise responses, whereas one study [21] investigated chronic adaptations following structured training. However, these findings should be interpreted cautiously given the limited available evidence.

Tosti et al. [18] reported attenuated lactate responses in breast cancer survivors compared with healthy controls during submaximal exercise sessions. The authors suggested that reduced carbohydrate oxidation and a greater reliance on fat oxidation may partly explain these findings. Reduced glycolytic demand during moderate-intensity exercise may limit lactate accumulation, particularly when oxidative metabolism predominates [26].

Neil et al. [20] observed lower lactate threshold power output in breast cancer survivors compared with comparator participants, suggesting altered exercise metabolism or reduced exercise tolerance. In contrast, Evans et al. [19] reported increased lactate responses following exercise, particularly immediately post-exercise and during recovery. These findings may reflect the influence of exercise intensity and the extent to which participants exceeded the lactate threshold during testing protocols.

Regarding chronic exercise exposure, Hiraoui et al. [21] demonstrated reduced resting lactate concentrations following a six-week cycling intervention. Chronic aerobic training may improve oxidative efficiency and metabolic flexibility, thereby reducing lactate accumulation during standardized exercise conditions and reflecting exercise adaptation [26].

Overall, these contrasting findings likely reflect differences in exercise intensity, intervention duration, metabolic conditioning, and comparator selection rather than true inconsistency in physiological response. Importantly, the included studies focused exclusively on systemic physiological responses to exercise rather than tumor-specific metabolic outcomes.

### 3.3. Potential Physiological Mechanisms

Several physiological mechanisms may contribute to differences in circulating lactate responses during exercise. Moderate and prolonged exercise may promote greater reliance on fat oxidation, reducing carbohydrate utilization and decreasing lactate production [27,28,29,30]. This metabolic adaptation is influenced by exercise intensity, hormonal regulation, and training status. In addition to production rates, circulating lactate responses are strongly influenced by lactate clearance and tissue reutilization capacity. Lactate produced during exercise may be oxidized by skeletal muscle, heart, liver, and other highly oxidative tissues through the lactate shuttle system. Exercise training can enhance mitochondrial oxidative capacity, monocarboxylate transporter activity, and enzymatic adaptations involved in lactate transport and utilization, thereby improving metabolic efficiency and reducing lactate accumulation during standardized exercise conditions.

Exercise intensity appears particularly relevant in determining lactate accumulation. During moderate-intensity exercise, reduced insulin secretion may facilitate increased fat oxidation and limit glycolytic dependence [31]. In contrast, high-intensity exercise relies predominantly on carbohydrate oxidation, increasing glycolytic flux and lactate production [32,33]. Consequently, variation in exercise intensity across the included studies may partly explain the heterogeneity of observed lactate responses.

These physiological considerations may help contextualize the variability of findings across studies, although they remain indirect interpretations rather than mechanisms directly tested within the included trials.

**Figure 5:**
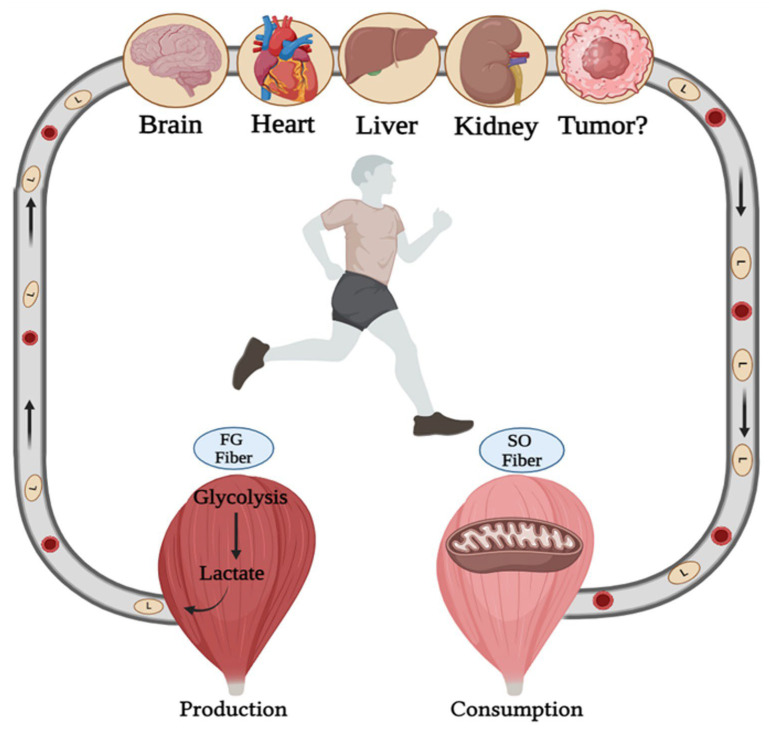
Conceptual representation of exercise-induced lactate production, systemic circulation, and tissue utilization during physical activity. Lactate produced by anaerobic glycolysis of skeletal muscle fibers during exercise may enter the bloodstream and be transported to oxidative tissues such as the heart, liver, kidney, and brain, where it can be utilized as a metabolic substrate. This physiological process is consistent with the lactate shuttle model. Potential interactions between circulating lactate and tumor metabolism remain speculative (based in in vitro studies) and have not been directly investigated in breast cancer survivors.

Figure 5 provides a conceptual overview of exercise-induced lactate dynamics during physical activity. Lactate generated by anaerobic glycolysis in muscle fibers may enter systemic circulation and subsequently be utilized by tissues that display a highly oxidative metabolism to obtain energy, including the heart, liver, kidney, and brain. This inter-organ lactate exchange reflects the lactate shuttle concept, whereby lactate functions not only as a metabolic byproduct but also as an energy substrate and signaling molecule involved in physiological adaptation [10,11,12,13]. Although circulating lactate may theoretically interact with tumor cells, the potential influence of circulating lactate on tumor cell metabolism remains unclear. Proposed mechanisms remain speculative and have not been directly evaluated in breast cancer survivors.

### 3.4. Mechanistic Cancer-Related Frameworks

The following mechanistic concepts are discussed exclusively as theoretical and preclinical frameworks intended to contextualize potential biological interactions between lactate metabolism and cancer biology. Importantly, none of these mechanisms were directly evaluated in the studies included in the present review, and therefore they should not be interpreted as mechanistic explanations supported by the current clinical findings.

Several biological mechanisms have been proposed to explain potential interactions between lactate metabolism, exercise, and tumor progression, including the Warburg effect, reverse Warburg effect, and AMPK/mTOR signaling pathways [15,32,33,34,35,36]. However, these concepts were not directly assessed in any of the studies included in the present review and should therefore be regarded as theoretical frameworks rather than explanations of the current findings. These pathways are included to provide biological context regarding potential links between exercise metabolism and cancer biology.

Lactate contributes to acidification of the tumor microenvironment and has been proposed as a metabolic substrate for energy production in adjacent tumor or stromal cells [32]. Altered glucose metabolism in cancer cells may influence signaling pathways such as AMPK and mTOR, both of which play central roles in cellular energy production and growth regulation [33,34,35].

The reverse Warburg effect describes a metabolic symbiosis in which stromal cells produce energy-rich metabolites that are subsequently utilized by cancer cells [36,37]. In this context, lactate may function as an intermediary metabolite within the tumor microenvironment. However, these mechanisms originate primarily from tissue-level (in vitro studies) and preclinical investigations and cannot be extrapolated to transient changes in peripheral blood lactate following exercise.

Accordingly, the mechanistic concepts discussed here are included solely to provide biological context and to identify future research directions rather than to explain the findings of the present meta-analysis.

### 3.5. Heterogeneity of Exercise Interventions and Outcome Comparability

A key methodological consideration in the present review is the substantial clinical and physiological heterogeneity across included exercise interventions. Studies differed with respect to exercise intensity, intervention duration, acute versus chronic exercise exposure, and comparator populations. These differences are particularly relevant in the context of lactate metabolism, as lactate production and clearance are highly dependent on exercise intensity and training status [26,31,32,33].

Acute maximal or near-maximal exercise protocols are expected to produce transient elevations in circulating lactate through increased glycolytic flux, whereas chronic aerobic training may reduce lactate accumulation because aerobic metabolism is dominant [29]. Consequently, pooling these physiologically distinct interventions into a single meta-analysis necessarily limits the interpretability of the pooled estimate.

Comparator heterogeneity represents an additional limitation. Some studies used healthy women as controls, whereas others used alternative breast cancer survivor comparator groups. Although the quantitative synthesis focused on within-breast cancer survivor exercise effects, these differences may suggest heterogeneous baseline physiological responses and complicate direct comparison across trials.

Although statistical heterogeneity was moderate (I^2^ = 31.1%), heterogeneity statistics are underpowered when few studies are available [25]. Therefore, the pooled effect estimate should be interpreted cautiously given the limited comparability across interventions and outcome assessments.

Similarly, comparability of lactate outcomes across trials remains limited. Included studies assessed lactate at different timepoints and under distinct physiological conditions, indicating different aspects of lactate metabolism rather than a unified experimental construct. Consequently, direct comparison of pooled lactate outcomes across studies remains inherently limited.

### 3.6. Leave-One-Out Sensitivity Analysis and Robustness of Findings

Additional support for the stability of the quantitative findings was provided by the leave-one-out sensitivity analysis. Sequential exclusion of individual studies did not materially alter the pooled effect estimate, indicating that no single trial disproportionately influenced the overall results. Although confidence intervals widened following exclusion of specific studies, pooled estimates consistently crossed the null value.

These results indicate that the absence of a statistically significant pooled effect reflects the consistency of the limited available evidence rather than reliance on any single dataset. Accordingly, the leave-one-out analysis supports the stability of the pooled estimate despite the small number of included studies and the physiological heterogeneity among interventions [25,36].

Given that only three studies were included in the meta-analysis, formal assessment of publication bias through funnel plots or statistical tests was not performed, as such methods lack sufficient power and interpretability [38,39]. Therefore, no conclusions regarding the presence or absence of publication bias can be drawn, and pooled estimates should be considered independently of these assessments.

### 3.7. Limitations and Strengths

This systematic review and meta-analysis has several important limitations. First, only a small number of randomized controlled trials met the eligibility criteria. Although four studies were included qualitatively, only three contributed to quantitative synthesis, limiting statistical power and precision. Consequently, statistical precision and robustness of pooled estimates remain limited [25]. An additional limitation is the absence of eligible randomized controlled trials published after 2019 despite the literature search extending through April 2026. This likely reflects the limited number of studies specifically evaluating exercise-induced circulating lactate responses in breast cancer survivors using randomized controlled trial methodology rather than an omission in the search process. Although broader exercise oncology studies were identified during screening, most did not assess circulating lactate outcomes or failed to meet predefined methodological eligibility criteria.

Second, methodological and clinical heterogeneity was substantial. Differences in exercise modality, intervention duration, lactate sampling protocols, exercise intensity, and comparator populations limit both interpretability and generalizability. Such variability limits direct comparability across studies and reduces confidence in pooled estimates [25].

An additional methodological limitation concerns the calculation of change-score variability. None of the included trials reported the standard deviation of change scores, requiring imputation using assumed pre–post correlation coefficients. Although sensitivity analyses using alternative plausible correlation values demonstrated relative stability in the direction of pooled estimates, precision remained sensitive to analytical assumptions. This issue is recognized in meta-analytic methodology when repeated-measure outcomes are synthesized without directly reported variance estimates [25].

Comparator heterogeneity across studies further limits interpretability. Healthy comparator groups and oncology-specific control groups may represent fundamentally different physiological contexts, which complicate pooled estimation and may influence baseline lactate dynamics, exercise tolerance, and metabolic responsiveness [18,19,20].

Despite these limitations, this review has several strengths. The study followed PRISMA guidelines [40], implemented a comprehensive search strategy across multiple databases, and applied validated methodological quality and risk-of-bias tools, including McMaster [22], PEDro [23], and Cochrane risk-of-bias criteria [24]. Furthermore, sensitivity analyses were conducted to examine the stability of pooled estimates, strengthening the transparency and robustness of the quantitative synthesis [25].

### 3.8. Contextual and Hypothesis-Generating Considerations

Given the limited and heterogeneous evidence base, any mechanistic interpretation remains speculative. Existing literature proposes several theoretical links between exercise, lactate metabolism, and cancer-related signaling pathways [15,16,17,27,28,29,30,31,32]. However, these concepts derive largely from preclinical models and narrative frameworks rather than direct evidence in breast cancer survivors.

In the context of the present review, these concepts are discussed exclusively to provide biological context and guide future research directions. The available evidence does not support causal claims, clinical implications, or mechanistic conclusions.

### 3.9. Future Directions and Implications for Sports Medicine

To our knowledge, this review represents the first attempt to synthesize evidence specifically examining exercise-induced circulating lactate responses in breast cancer survivors. Although no statistically significant pooled effect was observed, these findings should be interpreted strictly as descriptive and hypothesis-generating.

Importantly, none of the included studies examined lactate concentrations within tumor tissue. This represents a major gap in the literature and limits understanding of whether exercise-induced metabolic changes have relevance for tumor-specific lactate dynamics [16,17,32].

Future investigations should evaluate standardized exercise protocols, homogeneous comparator groups, and consistent lactate sampling procedures. Studies integrating tumor tissue biomarkers, imaging approaches, or mechanistic metabolic outcomes may provide greater insight into whether exercise-induced lactate responses are linked to tumor metabolism, angiogenic signaling, or cancer progression [32,33,34,35,36].

From a sports medicine perspective, understanding physiological metabolic responses to exercise may contribute to improved characterization of exercise tolerance and adaptation in breast cancer survivors. However, current evidence remains insufficient to support oncologic interpretations or clinical recommendations based on circulating lactate responses alone [2,26,41]. The absence of recent randomized controlled trials in this field further highlights the need for updated investigations using standardized metabolic outcomes and contemporary exercise oncology methodologies.

### 3.10. Practical Applications

Although the current evidence does not support the use of circulating lactate as a clinically meaningful oncologic biomarker, the findings of this review may still have practical relevance for exercise professionals involved in breast cancer rehabilitation. Exercise prescription in breast cancer survivors should be individualized according to treatment history, cardiorespiratory fitness, fatigue levels, comorbidities, and functional capacity.

Given the heterogeneity in exercise tolerance and metabolic responses observed across studies, exercise interventions should preferentially follow progressive and supervised approaches, particularly during the early phases of rehabilitation. Moderate-intensity aerobic exercise appears to induce physiological lactate responses without evidence of clinically adverse metabolic effects; however, current evidence remains insufficient to support lactate-guided exercise prescription in this population.

Accordingly, physicians, physiotherapists, exercise physiologists, and rehabilitation specialists should continue prioritizing established exercise prescription principles focused on safety, symptom management, physical function, and quality of life rather than relying on circulating lactate responses as a marker of oncologic adaptation or prognosis.

## 4. Materials and Methods

### 4.1. Protocol and Registration

This systematic review was conducted and reported in accordance with the Preferred Reporting Items for Systematic Reviews and Meta-Analyses (PRISMA) guidelines [40]. The review protocol was prospectively registered in the International Prospective Register of Systematic Reviews (PROSPERO; registration number CRD42024504288).

### 4.2. Information Sources and Search Strategy

A systematic literature search was performed in PubMed (Medline), Scopus, and Web of Science from database inception to April 23, 2026. The search strategy included combinations of terms related to breast cancer, exercise, lactate, and randomized controlled trials. Detailed search strategies for each database are provided in Supplementary Tables S1a–c. Briefly, the search strategy combined controlled vocabulary terms and free-text keywords related to four main domains: breast cancer, exercise or physical activity, lactate-related outcomes, and randomized controlled trial methodology.

No restrictions regarding publication date or language were applied during the search process. Unpublished studies were not included. Reference lists of eligible articles were manually screened to identify additional relevant studies not captured in the primary database search.

### 4.3. Eligibility Criteria

Eligibility criteria were defined according to the PICOS framework [42], including breast cancer survivor cohorts as the population (P), exercise training interventions as the intervention (I), non-exercise control groups or alternative comparator conditions within breast cancer survivor populations as the comparison (C), circulating blood lactate responses as the outcome (O), and randomized controlled trials as the study design (S). Studies involving healthy participants as comparator groups were also considered eligible; however, when appropriate, effect estimates were derived using within-group pre–post changes from the breast cancer survivor intervention arm. Because comparator groups varied across studies, including healthy controls and alternative breast cancer survivor cohorts, pooled quantitative analyses were preferentially based on within-group pre–post exercise changes in breast cancer survivor intervention groups when sufficient data were available. This approach was used to improve physiological comparability across heterogeneous comparator conditions.

### 4.4. Study Selection

Studies were included if they met the following criteria: (i) Randomized controlled trial design to ensure the highest level of evidence and minimize potential confounding inherent to observational or uncontrolled studies; (ii) Inclusion of breast cancer survivor populations; (iii) Reporting of circulating lactate outcomes or sufficient statistical information for effect size calculation; (iv) Use of original datasets when duplicate publications from the same cohort were identified.

Animal studies, observational studies, uncontrolled interventions, editorials, reviews, conference abstracts, notes, and other non-original publications were excluded.

The search strategy was designed to maximize sensitivity and capture studies evaluating exercise-related lactate responses in breast cancer populations. Search terms focused on four core domains: breast cancer, exercise or physical activity, lactate-related outcomes, and randomized controlled trial methodology.

Title/abstract screening and full-text eligibility assessment were independently performed by two reviewers using duplicate screening procedures.

### 4.5. Data Extraction

Data extraction included study characteristics, participant demographics, intervention details, comparator conditions, and lactate-related outcomes. Information collected from each eligible study included first author, publication year, country of origin, sample size, mean age, body mass index (BMI), cancer stage, intervention duration, exercise protocol characteristics, and lactate outcome measures.

A standardized data extraction form was developed and used to ensure consistency across reviewers during the extraction process.

When reported data were incomplete or unclear, attempts were made to contact corresponding authors for clarification. Two reviewers independently performed study selection and data extraction using duplicate procedures within Covidence systematic review software (Veritas Health Innovation, Melbourne, Australia. Available at www.covidence.org). Any discrepancies were resolved through discussion, with consultation from a third reviewer when necessary.

Formal inter-rater agreement statistics (e.g., Cohen’s kappa) were not calculated because of the limited number of included studies; however, reviewer agreement procedures were applied consistently throughout the review process to ensure methodological rigor and transparency.

### 4.6. Methodological Quality Assessment and Risk of Bias

Methodological quality was assessed using the Physiotherapy Evidence Database (PEDro) scale [23] and the McMaster Critical Review Form for Quantitative Studies [22]. These instruments are widely used for evaluating methodological rigor, internal validity, and reporting quality in rehabilitation and clinical intervention studies.

Risk of bias was independently evaluated by two reviewers using the Cochrane Risk of Bias tool for randomized controlled trials [24]. Disagreements were resolved through discussion or consultation with a third reviewer.

Given the small number of included studies, formal inter-rater reliability statistics were not calculated. However, standardized methodological tools were applied consistently to ensure transparent and reproducible quality assessment.

For risk-of-bias evaluation, studies were categorized as low risk, some concerns, or high risk depending on the presence of methodological limitations that could influence reported outcomes [24].

PEDro and McMaster assessments were used to evaluate methodological and reporting quality, whereas the Cochrane risk-of-bias tool specifically assessed potential sources of bias affecting internal validity. Because these instruments evaluate different methodological constructs, results were interpreted complementarily rather than combined into a single overall quality score.

### 4.7. Statistical Analysis

Meta-analysis was performed using Stata software version 14 (StataCorp, College Station, TX, USA). Statistical significance was defined as *p* ≤ 0.05.

Pooled effect estimates were calculated using mean changes in circulating lactate concentrations and their corresponding standard deviations. Because none of the included studies reported standard deviations for change scores, *SD_change* values were estimated using a standard pre–post correlation formula based on pre-intervention and post-intervention variability [25].

$$SD_{change}=\sqrt{SD_{pre}^{2}+SD_{post}^{2}-2r\cdot SD_{pre}\cdot SD_{post}}$$

A pre–post correlation coefficient (r) of 0.8 was assumed based on methodological recommendations for repeated physiological measurements in intervention-based studies [25].

A random-effects model was applied to account for expected study heterogeneity and variability in intervention characteristics [25]. Statistical heterogeneity was assessed using Cochran’s Q test and the I^2^ statistic, with I^2^ values greater than 50% indicating substantial heterogeneity [25].

Sensitivity analyses were performed using a leave-one-out approach to evaluate the influence of individual studies on pooled estimates. Additional sensitivity analyses were conducted using alternative pre–post correlation coefficients (r = 0.5 and r = 0.7) to evaluate the influence of *SD_change* assumptions on pooled results [25].

Publication bias was explored through funnel plot inspection and Egger’s and Begg’s statistical tests [38,39]. However, given the small number of included studies, publication bias findings were interpreted cautiously because statistical power is limited when few studies are available [38,39].

Due to heterogeneity in exercise modality, comparator conditions, and lactate assessment timing, the quantitative synthesis was considered exploratory and intended to summarize physiological response patterns rather than establish definitive effect estimates.

## 5. Conclusions

Current evidence suggests that exercise-induced changes in circulating lactate concentrations in breast cancer survivors are small, inconsistently reported, and derived from a limited and heterogeneous evidence base. No statistically significant pooled effect was identified; however, the available data remain insufficient to support definitive conclusions regarding the physiological magnitude or clinical relevance of circulating lactate responses following exercise interventions.

Importantly, circulating blood lactate cannot currently be considered a validated biomarker of oncologic prognosis, recurrence risk, or tumor progression. Therefore, the present findings should be interpreted as exploratory and hypothesis-generating rather than clinically actionable.

The primary contribution of this review lies in defining current evidentiary boundaries and identifying key research gaps. Future studies using standardized exercise protocols, harmonized lactate assessment procedures, and mechanistic or longitudinal designs are needed to clarify whether exercise-related lactate dynamics have relevance for breast cancer survivorship.

## Figures and Tables

**Table 2:** Methodological quality assessment of included studies using the PEDro scale.

Study, Year	Items											Total	%	Quality Score
	1	2	3	4	5	6	7	8	9	10	11			
Tosti et al. (2011) [18]	1	1	1	1	0	0	0	1	1	1	1	8	72.7	G
Neil et al. (2013) [20]	1	1	1	1	0	1	0	1	1	1	1	9	81.8	E
Evans et al. (2016) [19]	1	1	1	1	0	0	0	1	1	1	1	8	72.7	G
Hiraoui et al. (2019) [21]	1	1	1	1	0	1	0	1	1	1	1	9	81.8	E

Abbreviations: E, excellent; G, good. Detailed item descriptions are available in the original PEDro publication [23].

**Table 3:** (A) Characteristics of randomized controlled trials included in the qualitative synthesis. Studies contributing to quantitative synthesis are indicated. (B) Exercise intervention characteristics and comparator conditions of studies included in the quantitative synthesis.

(**A**)						
**Study**	**Design**	**Participants (*n*)**	**Age (years) Mean ± SD**	**BMI (kg/m^2^) Mean ± SD**	**Cancer Stage**	**Included in Meta-Analysis**
Tosti et al. (2011) [18]	RCT	CG: 7/ING: 7	CG: 50.9 ± 3.6/ING: 50.6 ± 3.3	CG: 28.2 ± 1.9/ING: 29.4 ± 1.1	I–III	Yes
Neil et al. (2013) [20]	RCT	CG: 11/ING: 16	CG: 56.8 ± 10.6/ING: 52.1 ± 7.9	CG: 20.5 ± 2.2/ING: 20.7 ± 1.7	I–IIIA	Yes
Evans et al. (2016) [19]	RCT	CG: 9/ING: 20	CG: 59 ± 5/ING: 49.7 ± 5.4	CG: 29.0 ± 4.6/ING: 28.4 ± 2.6	I–III	Yes
Hiraoui et al. (2019) [21]	RCT	CG: 12/ING: 20	CG: 48.9 ± 4.8	CG: 27.6 ± 2.6	I–IIIA	No *
(**B**)						
**Study**	**Exercise Protocol**		**Duration**	**Comparator**	**Main Findings**	
Tosti et al. (2011) [18]	Cycle ergometer session including warm-up, steady-state cycling, and cooldown		Single session	Healthy women	Lower lactate response observed in breast cancer survivors compared with controls	
Neil et al. (2013) [20]	Maximal cycle ergometer exercise test		Single session	BCS without persistent fatigue	No significant differences in lactate response	
Evans et al. (2016) [19]	30 min cycling at 60% VO_2_peak		Single session	Healthy women	Attenuated lactate response in breast cancer survivors; larger glucose and FFA changes	

Abbreviations: BCS, breast cancer survivors; FFA, free fatty acids; VO_2_peak, peak oxygen uptake; BMI, body mass index; CG, control group; ING, intervention group; RCT, randomized controlled trial; SD, standard deviation. (*) Study excluded from quantitative synthesis because outcome variability required for effect size estimation was not reported.

**Table 4:** Baseline, post-intervention, and change values for circulating blood lactate concentrations across included studies.

Study	Group	*n*	Pre-Intervention (mmol/L) Mean ± SD	Post-Intervention (mmol/L) Mean ± SD	Change (mmol/L) Mean ± SD
Tosti et al. (2011) [18]	CG	7	NR	NR	+3.83 ± 6.56
	ING	7	NR	NR	+2.23 ± 4.36
Neil et al. (2013) [20]	CG	11	1.1 ± 0.3	2.1 ± 1.0	+1.0 ± 0.78
	ING	16	1.2 ± 0.6	1.9 ± 0.5	+0.7 ± 0.36
Evans et al. (2016) [19]	CG	9	NR	NR	+1.74 ± 0.35
	ING	9	NR	NR	+1.93 ± 0.37
Hiraoui et al. (2019) [21]	CG	12	2.69 ± 0.19	2.60 ± 0.13	−0.09 ± 0.11
	ING	20	1.94 ± 0.46	1.47 ± 0.36	−0.47 ± 0.27

Abbreviations: CG, control group; ING, intervention group; NR, not reported; SD, standard deviation. Note: When pre- and post-intervention values were unavailable, change scores were derived from reported summary statistics as described in the Methods section.

**Table 5:** Leave-one-out sensitivity analysis evaluating the influence of individual studies on pooled weighted mean differences (WMDs) for circulating lactate outcomes. Sequential exclusion of each study did not materially alter the direction or magnitude of the pooled estimate.

Study Removed	Pooled WMD (95% CI)
None (overall model)	0.03 (−0.24 to 0.31)
Tosti et al. (2011) [18] removed	−0.02 (−0.50 to 0.46)
Neil et al. (2013) [20] removed	0.18 (−0.15 to 0.51)
Evans et al. (2016) [19] removed	−0.31 (−0.80 to 0.18)

Abbreviations: CI, confidence interval; WMD, weighted mean difference.

## Data Availability

The original contributions presented in this study are included in the article/Supplementary Materials. Further inquiries can be directed to the corresponding authors.

## Supplementary Materials

The following supporting information can be downloaded at: https://www.mdpi.com/article/10.3390/muscles5030047/s1, Table S1a: Search strategy used in PubMed (MEDLINE); Table S1b: Search strategy used in Web of Science; Table S1c: Search Strategy used in Scopus.
