# Supplementary material for: Exercise-Induced Circulating Lactate Responses in Breast Cancer Survivors: A Systematic Review and Exploratory Meta-Analysis
Source: Muscles. 2026 Jul 2;5(3):47. doi: 10.3390/muscles5030047 (PMC13398076; doi:10.3390/muscles5030047)
Supplement: Supplementary file 1 [file muscles-05-00047-s001.zip › muscles-4325613-supplementary.pdf]

Supplementary Material

Supplementary Table S1a. Search strategy used in PubMed (MEDLINE).

**Category**	**Search Terms**
**1**	Exercise-related terms: ("Exercise"[MeSH] OR exercise OR "physical activity" OR sport OR fitness OR "aerobic exercise" OR "endurance exercise" OR "resistance exercise" OR "strength exercise" OR "aerobic training" OR "endurance training" OR "resistance training" OR "strength training")
**2**	Study design terms: ("randomized controlled trial"[Publication Type] OR "controlled clinical trial"[Publication Type] OR randomized OR randomised OR randomly)
**3**	Breast cancer terms: ("Breast Neoplasms"[MeSH] OR "breast cancer" OR "breast neoplasm*" OR "breast carcinoma*" OR "breast tumour*" OR "breast tumor*" OR "mammary cancer*" OR "mammary neoplasm*" OR "mammary carcinoma*")
**4**	Lactate-related terms: (lactate OR "lactic acid" OR "blood lactate")
**5 Final search strategy:**	1 AND 2 AND 3 AND 4

Supplementary Table S1b. Search strategy used in Web of Science.

**Category**	**Search Terms**
**1**	exercise OR "physical activity" OR sport* OR fitness OR "aerobic exercise*" OR "endurance exercise*" OR "resistance exercise*" OR "strength exercise*" OR "aerobic training*" OR "endurance training*" OR "resistance training*" OR "strength training*"
**2**	"randomized controlled trial" OR "controlled clinical trial" OR randomized OR randomised OR randomly
**3**	"breast cancer" OR "breast neoplasm*" OR "breast carcinoma*" OR "breast tumour*" OR "breast tumor*" OR "mammary cancer*" OR "mammary neoplasm*" OR "mammary carcinoma*"
**4**	“lactate OR "lactic acid" OR "blood lactate"

**5 Final search strategy:**	1 AND 2 AND 3 AND 4

Supplementary Table S1c. Search strategy used in Scopus.

**Category**	**Search Terms**
**1**	exercise OR "physical activity" OR sport OR fitness OR "aerobic exercise" OR "endurance exercise" OR "resistance exercise" OR "strength exercise" OR "aerobic training" OR "endurance training" OR "resistance training" OR "strength training"
**2**	"randomized controlled trial" OR "controlled clinical trial" OR randomized OR randomised OR randomly
**3**	"breast cancer" OR "breast neoplasm*" OR "breast carcinoma*" OR "breast tumour*" OR "breast tumor*" OR "mammary cancer*" OR "mammary neoplasm*" OR "mammary carcinoma*"
**4**	lactate OR "lactic acid" OR "blood lactate"
**5 Final search strategy:**	1 AND 2 AND 3 AND 4
